# Tuning up microbiome analysis to monitor WWTPs’ biological reactors functioning

**DOI:** 10.1038/s41598-020-61092-1

**Published:** 2020-03-05

**Authors:** Miguel de Celis, Ignacio Belda, Rüdiger Ortiz-Álvarez, Lucía Arregui, Domingo Marquina, Susana Serrano, Antonio Santos

**Affiliations:** 1 Department of Genetics, Physiology and Microbiology, - Unit of Microbiology, Complutense University of Madrid, 28040 Madrid, Spain; 2 Department of Biology, Geology, Physics and Inorganic Chemistry - Area of Biodiversity and Conservation, Rey Juan Carlos University, 28933 Móstoles, Spain; 3 Integrative Freshwater Ecology Group, Center for Advanced Studies of Blanes (CEAB - CSIC), 17300 Blanes, Catalonia Spain

**Keywords:** Applied microbiology, Bacteria, Microbial communities, Microbial ecology, Water microbiology

## Abstract

Wastewater treatment plants (WWTPs) are necessary to protect ecosystems quality and human health. Their function relies on the degradation of organic matter and nutrients from a water influent, prior to the effluent release into the environment. In this work we studied the bacterial community dynamics of a municipal WWTP with a membrane bioreactor through 16S rRNA gene sequencing. The main phyla identified in the wastewater were *Proteobacteria*, *Bacteroidetes*, *Chloroflexi*, *Planctomycetes* and *Actinobacteria*. The WWTP is located in Spain and, like other studied WWTP in temperate climate zones, the temperature played a major role in community assembly. Seasonal community succession is observed along the two years sampling period, in addition to a continual annual drift in the microbial populations. The core community of the WWTP bioreactor was also studied, where a small fraction of sequence variants constituted a large fraction of the total abundance. This core microbiome stability along the sampling period and the likewise dissimilarity patterns along the temperature gradient makes this feature a good candidate for a new process control in WWTPs.

## Introduction

Contaminant removal from industrial and urban wastewater is a capital issue for the protection of the natural environment and human health. Most Wastewater Treatment Plants (WWTPs) rely on conventional biological treatment systems, such as activated sludge processes. In combination with different redox conditions (anaerobic, anoxic and aerobic), these biological processes assure the removal of organic matter and nutrients^1^. As the most common biological wastewater treatment application, activated sludges are complex microbial ecosystems composed of Bacteria, Archaea, Eukarya and viruses^2,3^. Recently, Membrane Bioreactor (MBR) technology in wastewater treatment has become a common practice globally. This process combines efficient biological degradation by activated sludge with a direct solid-liquid membrane separation, offering several advantages over other systems such as a superior effluent quality, high biodegradation capacity and low sludge production^4,5^. Developed activated sludge heavily depends on its microbial populations for the removal of nutrients and organic pollutants and hence, the depuration performance of WWTPs. Understanding the composition, structure and functioning of these microbial communities is essential for constructing improved wastewater treatment plants^6^.

Activated sludge bacterial communities have been widely studied either by culture-dependent methods or by molecular approaches revealing, on one side a high bacterial diversity, on the other the fact that a small fraction of taxa accounted for the majority of total abundance^7,8^. In previous works, *Proteobacteria* was the most abundant phylum in WWTPs (accounting for 30–80% of the total abundance), followed by *Bacteroidetes*, *Firmicutes* and *Actinobacteria*^9–13^. These communities include taxa involved in different metabolic pathways (nitrogen fixation, nitrification, denitrification, sulphur oxidation, etc.); physiological groups like anaerobic, aerobic, phototrophic, heterotrophic, etc.; and inter-species relationships systems (i.e. quorum sensing)^14^. Furthermore, microbial populations inhabiting WWTP bioreactors can differ among different stations and over time, and their biodiversity is thought to play an important role in enabling and facilitating particular ecosystem functions, such as nutrients removal. Predicting the behaviour of particular populations and communities under different situations and how these are linked to the performance of a particular ecosystem process, will improve the efficiency of critical processes in WWTP communities^15^.

WWTP performance should guarantee a certain effluent quality which will vary according to the posterior uses of water. Different techniques have been developed to measure activated sludge quality, such as Sludge Biotic Index (based on the presence and abundance of certain protists) or the Sludge Index (considering macroscopic and microscopic activated sludge features)^16–18^. A complementary tool to control microbial community and activated sludge stability based on the bacterial populations is necessary to assess WWTP performance and prevent activated sludge disruptions. High-throughput sequencing (HTS) of conserved regions in microbial genomes (i.e. bacterial 16S rRNA), are nowadays considered the most reliable and cost-effective method to study the microbial composition and ecological dynamics of WWTPs^2,19^.

The seasonality of natural ecosystems such as oceans, freshwater or soils has been widely studied with HTS, but little attention has been given to anthropic ecosystems. Indeed, in the activated sludge microbial community, seasonal dynamics may affect the performance and stability of organic material and nutrient removal (nitrogen and phosphorus) in WWTPs^8,20^. This aspect should be studied in more detail with the aim of using tag-sequencing as routine technology as a means of learning about microbial ecology in WWTPs. Furthermore, it is necessary to understand the dynamics of microbial communities and their seasonal variations for predicting undesirable changes in the functional diversity of activated sludges, for a better control over operational parameters of WWTPs.

In this work, we have studied monthly the microbial community dynamics of the three reactors of a full-scale municipal WWTP located in North-East of Spain over a period of 2 years. We have defined the core microbiome of this WWTP, its inter-reactor variability and its evolution along time. The results showed a seasonal community variation observed in the full bacterial community that does not exist in the core microbiome. The core microbiome preserved a narrower range of stability while displaying the annual drift trend, making it a good candidate to be considered as a new WWTP process control indicator.

## Results

### Bacterial community composition

A total of 66 mixed liquor samples were analysed from a full scale MBR-WWTP. Samples were taken monthly along two years in the three compartments of the reactor, allowing the evaluation of annual community variability. A total of 3,454,577 good quality 16S rRNA sequences were obtained, resulting in 6,245 Amplicon Sequence Variants (ASVs). ASVs methods allow the distinction of sequences differing by as little as one nucleotide^21^.

**Figure 1:**
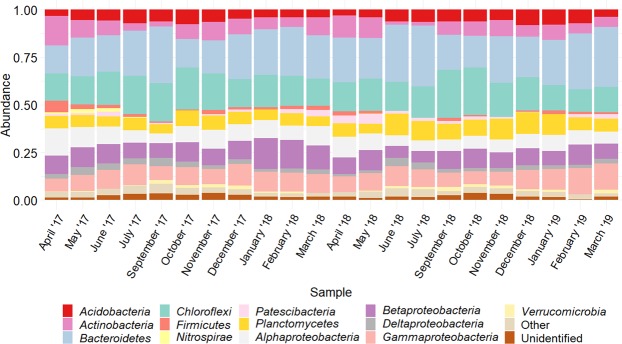
Mean relative abundance of dominant bacterial phyla with a frequency higher than 1% on at least one sample. “Other”, includes phyla of frequency <1%; and “unidentified”, taxonomically unassigned taxa. Sampling months are indicated, starting April 2017 and ending March 2019. There was no sampling in August. Phylum *Proteobacteria* is shown subdivided in classes *Alpha-*, *Beta-*, *Delta-* and *Gammaproteobacteria*.

Taxonomic assignation against SILVA (release 132) allowed the identification of 39 phyla. We considered as main phyla those whose abundance was higher than 1% on at least one sample (Fig. 1). The average relative abundances, of sequence reads for the main phyla in all stages were as follows: *Proteobacteria* (considering *Alpha-, Beta-, Delta-* and *Gammaproteobacteria*) 30.18% ± 3.84%, *Bacteroidetes* 23.12% ± 5.12%, *Chloroflexi* 17.15% ± 3.55%, *Planctomycetes* 7.53% ± 2.10%, *Actinobacteria* 7.20% ± 3.25%, *Acidobacteria* 5.63% ± 1.52%, *Patescibacteria* 1.74% ± 1.17%, *Firmicutes* 1.66% ± 1.11%, *Verrucomicrobia* 1.25% ± 0.44%, *Nitrospirae* 0.51% ± 0.54%. All those phyla, except *Nitrospirae*, that is only present in spring and summer samples, tended to be persistent over the 2-year sampling period. The phylum *Proteobacteria* is more abundant in winter and spring samples, and is mainly represented by the classes *Alpha* (8.21% ± 2.49%), *Beta-* (9.71% ± 3.12%), *Delta-* (2.55% ± 1.06%) and *Gammaproteobacteria* (9.52% ± 2.21%). However, other phyla like *Bacteroidetes*, *Chloroflexi* or *Planctomycetes* were less abundant during these months (p < 0.05). This trend shows a seasonal distribution of key phyla in mixed liquors. Average relative abundance of *Archaea* was 0.25% ± 0.20%, being the phylum *Euryarchaeota* the most abundant (0.21% ± 0.13%), thus, those populations were not considered in subsequent analysis.

### Diversity, seasonal dynamics and functional predictions of the wastewater microbiome

**Figure 2:**
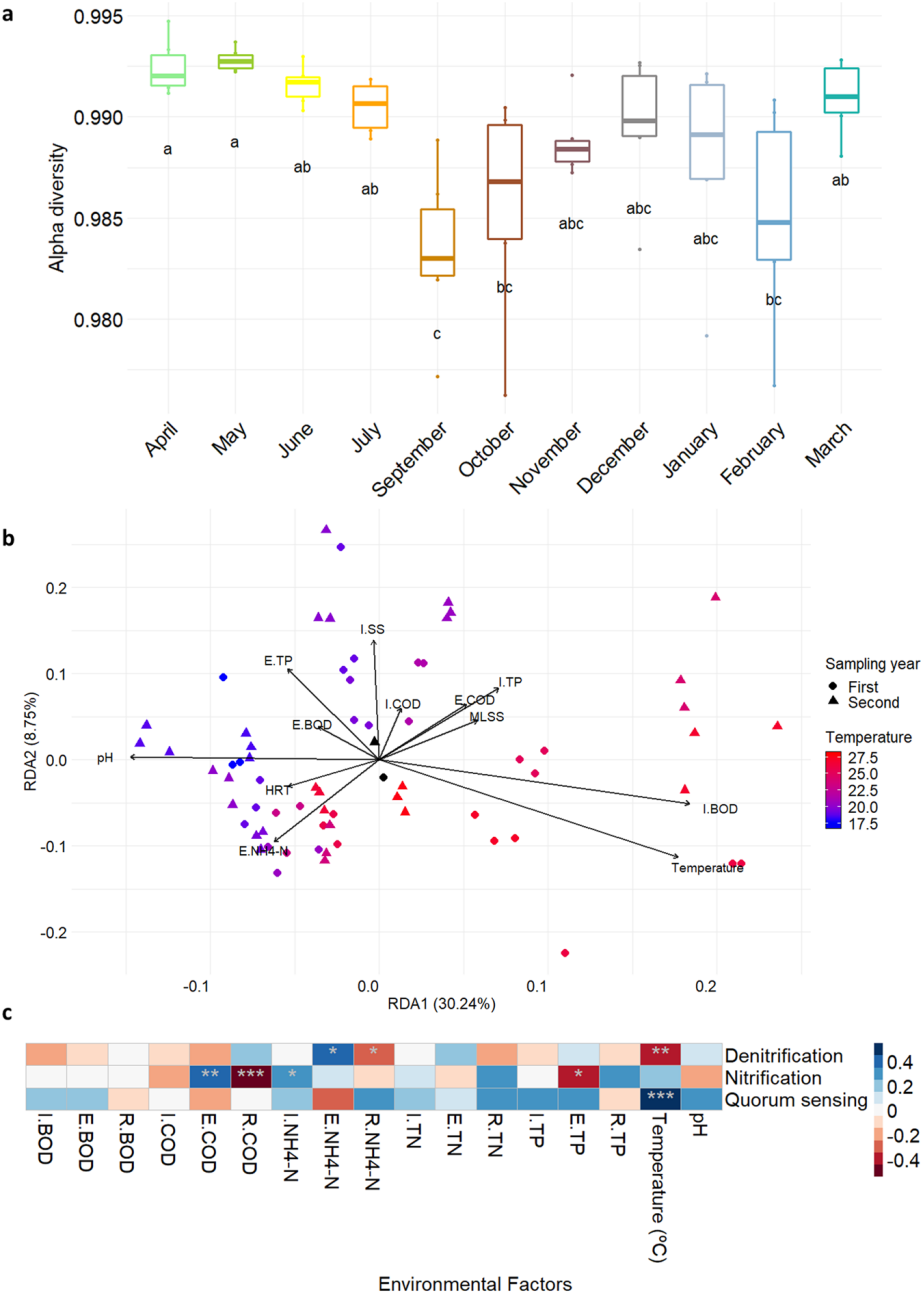
Microbial diversity analysis. (**a**) Simpson diversity index as a measurement of alpha diversity. An ANOVA test and a LSD (Least Square Difference) test were conducted (**a**–**c** indicate significance groups). (**b**) Redundancy analysis (RDA). Dots and triangles correspond with each sample on the first or second sampling year, respectively. Black points stand for the centroid of the first and second sampling year. Colour is indicative of the temperature gradient, and lines show the physical-chemical factors constrained into the ordination. (**c**) pairs between predicted functional groups (Supplementary Table S2), and physical-chemical factors. Dark blue indicates stronger positive correlations and dark red stronger negative correlations. Asterisks denote the significance levels (***p < 0.001, **p < 0.01 and *p < 0.05). P values were adjusted for multiple testing using the Bonferroni correction.

To understand the annual variations found in the WWTP, the microbial community diversity (alpha diversity) was calculated, using the Simpson diversity index (D’). No differences were found in the communities inhabiting each compartment of the bioreactor (tested by analysis of similarity, ANOSIM, p = 0.975), so further statistical analysis was performed considering the sampling time. September, October and February were the months with lower diversity, while April and May were the months with higher diversity (Fig. 2a). Overall, autumn appeared to be the season with the lower alpha diversity values, popping a rebound during spring months.

The multidimensional ordination (NMDS) of the sequence data based on Bray-Curtis dissimilarities was used to compare the ASV profiles of communities (Supplementary Fig. S1). This analysis revealed seasonal clustering of communities, where samples were sorted monthly, from April 2017 (first sample) to March 2019 (last sample), showing an annual cycle in both years (Supplementary Fig. S1). Despite that the annual cycle is repeated in both sampling years, permutational multivariate analysis of variance (PERMANOVA) test showed significant differences between them in terms of community structure (p = 0.001). PERMANOVA tests were also used to study the effect of physical-chemical parameters on the bacterial community, identifying the temperature of the bioreactor as the principal factor impacting microbial populations (R^2^ = 0.162, p = 0.001). The significant parameters (p < 0.01), along with temperature, explained a 54.95% of the variation observed between samples. Figure 2b shows a redundancy analysis (RDA) where some constrained features (influent biochemical oxygen demand-I.BOD) contributes to the first component in the same direction than temperature, as the most relevant variable, and some others (pH, effluent ammonia concentration-E.NH_4_-N, and hydraulic retention time-HRT) contributes in an opposite direction in shaping bacterial communities composition.

Through a functional prediction based on 16S rRNA sequences, it is possible to estimate functional traits of the bacterial population in the wastewater. We analysed correlations of 57 environmental functions from 5 pathways (involved in nitrogen and phosphorous metabolism, biodegradation and quorum sensing) and the wastewater physical-chemical factors (Fig. 2c). The temperature was significantly correlated with the presence of quorum sensing related genes (p < 0.001) and inversely correlated with denitrification (p < 0.01). Nitrification was significantly correlated with chemical oxygen demand (COD) in the effluent (p < 0.01) and its reduction (inversely, p < 0.001).

### Wastewater core bacterial community

**Figure 3:**
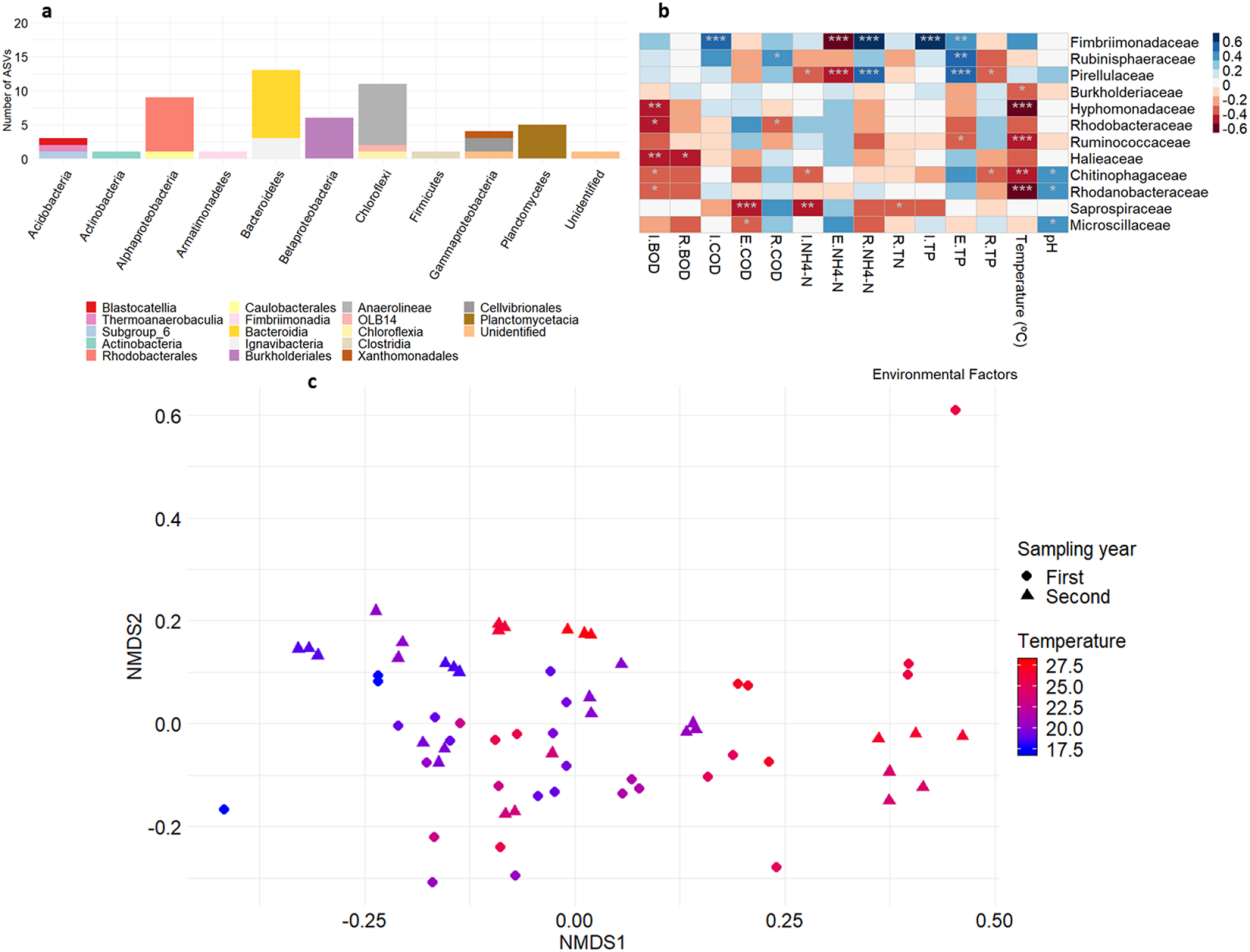
Composition, functional correlations and multidimensional ordination based on core ASVs in the activated sludge samples. (**a**) Taxonomic composition of core ASVs at phylum level and class level. *Proteobacteria* group is divided in inferior taxonomic levels. (**b**) Spearman’s rank correlation coefficient between core ASVs, at family level assignation, and physical-chemical factors. Only core families and physical-chemical parameters with at least one significative correlation are shown. (**c**) Beta diversity of core community based on a non-metric multidimensional scaling analysis (NMDS, stress = 0.152).

The core bacterial community was defined based on the high occurrence frequency of the ASVs. About 0.88% of total ASVs (55 ASVs) were present in 100% of the activated sludge samples and constituted a core that accounted for 36.5 ± 5.2% of the sequence reads. Most of the core community members belonged to *Alpha-*, *Beta-* and *Gammaproteobacteria* classes; and *Bacteroidetes* and *Chloroflexi* phyla (Fig. 3a). Spearman’s rank correlation coefficient revealed that changes of activated sludge community composition, at the family level, were significantly correlated with physical-chemical parameters. Out of the 30 families that form the core microbiome, 12 were significantly correlated (adjusted p < 0.05) to at least one physical-chemical parameter (Fig. 3b). Ammonia removal is correlated with *Pirellulaceae* and *Fimbriimonadaceae* families (p < 0.001), while temperature of the bioreactor is inversely correlated with *Burkholderiaceae*, *Hyphomonadaceae*, *Ruminococcaceae*, *Chitinophagaceae* and *Rhodanobacteraceae* families (p < 0.05).

The beta diversity of the core community showed a clear clustering and evolution based on the temperature of the sample (PERMANOVA test, R^2^ = 0.176, p = 0.001) (Fig. 3c). As occurred with the beta-diversity of the full community, PERMANOVA test showed a significant difference between the core microbiome composition the two years sampled (R^2^ = 0.053, p = 0.001).

**Figure 4:**
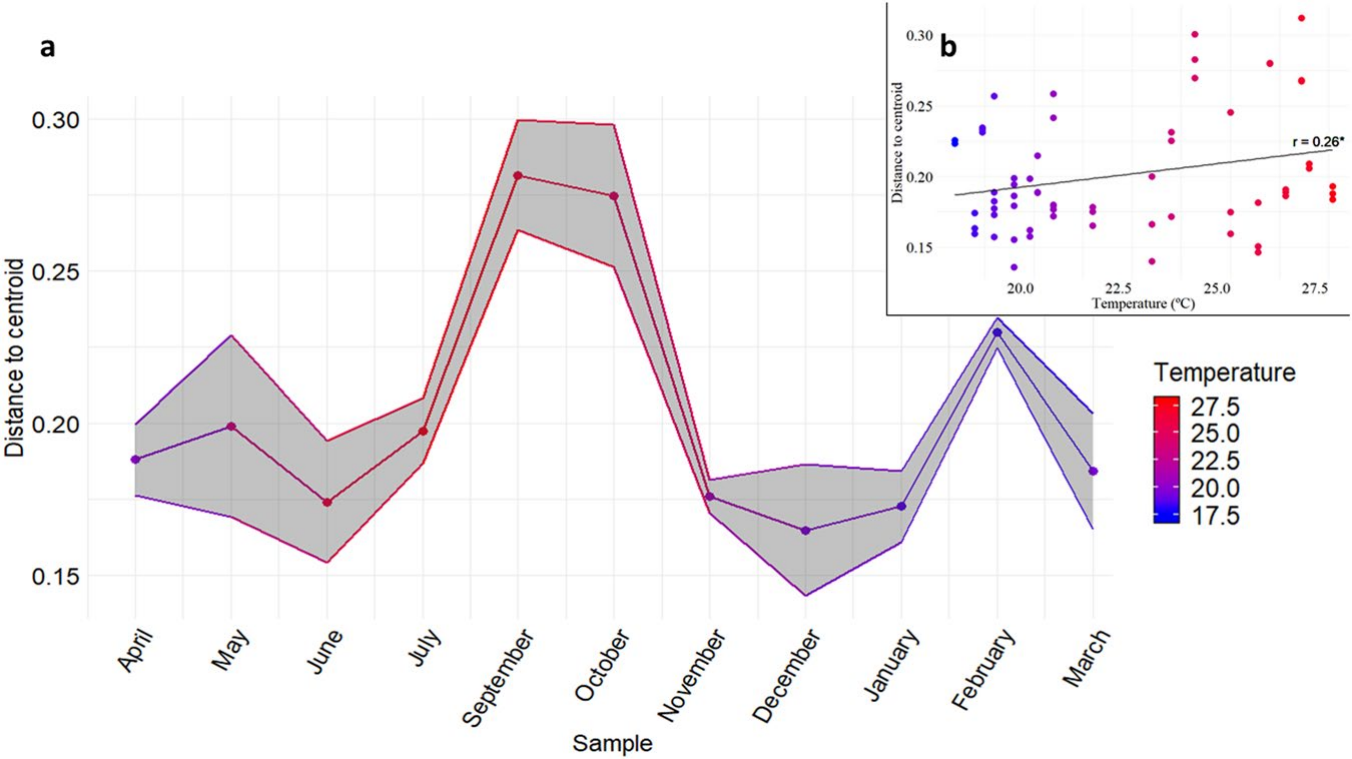
Mean distance to centroid of bacterial population dissimilarities of samples taken on each sampled month, accounting for both sampling years. (**a**) Mean distances are represented along the sampling months. (**b**) Correlation between mean distances and temperature (r = 0.26, p = 0.04). Each sampling year were considered independent, and both centroids were calculated. The colour gradient shows the average monthly temperature (°C) on both sampling years. For a desegregate visualization of the two year sampling monitoring see Supplementary Fig. S3.

Indeed, when calculating the distance of each sample to their sampling year centroid (Fig. 4) we observed that when the average temperatures rose, these distances also increased. This comparison indicated that the higher the temperature the higher the changes in the core bacterial community. Both years presented a similar pattern of temperature/community dispersion, making it possible to define a new system stability measure (Supplementary Fig. S2).

## Discussion

In this work, we studied the bacterial community dynamics of a municipal waste water treatment plant with a membrane bioreactor (MBR-WWTP). This bioreactor is functionally divided into three sections, attending to their oxygenation conditions (anoxic, oxic-anoxic, anoxic); besides it has a recirculation system connecting the third stage with the first. Through 16S rRNA gene sequencing we identified *Proteobacteria*, *Bacteroidetes*, *Chloroflexi*, *Planctomycetes* and *Actinobacteria* as the main phyla in the bacterial community studied, making 85.17% ± 2.43% of the total sequence reads. The most common phyla found in WWTPs around the globe are *Proteobacteria* and *Bacteroidetes*. The two *Proteobacteria* most common families in the WWTP studied were *Burkholderiaceae* (*Betaproteobacteria*), that has members known to degrade PCBs^22^; and *Rhodobacteraceae* (*Alphaproteobacteria*), involved in sulfur and carbon cycles^23^. Within *Bacteroidetes* phyla, the most abundant families were *Chitinophagaceae*, aerobic heterotrophs degraders of organic matter^24^; and *Saprospiraceae* which includes many bacteria associated with protein hydrolysis and epiphytic bacteria attached on some filamentous bacteria (Supplementary Fig. S3)^25^. In addition, the core bacterial community was determined based on the occurrence of sequence variants (ASVs) in the samples. Despite consisting in a relatively low number of ASVs (55 or 0.88% of the total), these accounted for a 36.5% of the total abundance, implying a hyper-dominance pattern similar to that found in a global WWTP core bacterial community study^8^.

Recent studies suggest that activated sludge microbial communities are shaped by deterministic (environmental and interspecies relationships) and neutral or stochastic (random events like colonization/extinction causing microbial dispersal) factors^26,27^. In natural aquatic ecosystems, a seasonal succession of the community is normally found, however, it is not as common to occur in artificial ecosystems^28^. Interestingly, we have found that in activated sludge systems the seasonal dynamics of microbial communities greatly affect the performance and stability of pollutant removal, and the main deterministic factor for this community dynamic in temperate climatic zones is the seasonal temperature fluctuation^15,20^. In the WWTP studied, samples were significantly different from each other, although, the samples of the same month of different years were more similar between them than with the other (Supplementary Fig. S1). This suggests a seasonal community succession but continual annual drift to the activated sludge at the studied facility, since both sampling years are significantly different (p = 0.001). The functional estimation performed in this work suggest a notable impact of temperature in certain bacterial-related functions such as a decrease in the presence of genes related with denitrification or a notable increase in those genes related with quorum sensing (Fig. 2c). This is of particular interest as quorum sensing regulates different metabolic mechanisms and coordinated behaviours at a community level; some of them of particular interest in WWTPs such as bacterial granulation and the maintenance of granular structures, or the undesired biofilm formation in MBR membranes^29,30^. This aspect should be studied in more detail for a better understanding of the impact of temperature in quorum sensing-mediated metabolisms in WWTPs.

As we have seen, the deterministic factors present in the studied WWTP greatly affects the bacterial community structure of its activated sludge (54.95% of the difference is explained by the physical-chemical parameters studied), with the temperature of the bioreactor as the main factor. Giving special attention to the families that compose the core microbiome, we observed that the ammonia removal is significantly correlated with the *Fimbriimonadaceae* and *Pirellulaceae* families. *Pirellulaceae* are ammonia-oxidizing bacteria^31^, while *Fimbriimonadaceae* belong to the *Armatinomonadetes* phylum, detected in ANAMMOX (ANaerobic AMMonium OXidation) consortia^32^. The two families aforementioned are positively correlated (p < 0.001, Spearman’s rank correlation coefficient = 0.495), implying that the *Fimbriimonadaceae* family either contains ammonia oxidizing taxons or has positive interactions with ammonia oxidizing bacteria, favouring the ammonia oxidizing processes. As we can see, bacteria present in the core microbiome are essential in the pollutant removal processes carried out in the activated sludge. Further research is necessary to study the biological interactions between both clades.

Nutrient and organic matter removal processes carried out in the activated sludge are based on the activity of the studied microorganisms, thus maintaining optimal conditions for their growth and development is necessary for a better process control and management^18^. The dynamics and organization of microbial communities, along with biotic components of the activated sludge, determine its quality and the efficiency of the depuration process^16^. Based on this premise, the operation of the reactor is currently controlled through various indicator organisms and various indices to assess the quality of the active sludge. These types of indices offer invaluable information about the effluent characteristics and the adequacy of the operational conditions^33^. Previous studies suggest that highly diverse ecosystems are more stable, due to the presence of species able to adapt to a changing ecosystem. The maintenance of stable activated sludge ecosystem requires understanding the functional stability of the bacterial communities^34^. These species representing the core microbiome are essential for wastewater treatment processes^35^. In addition, MBR systems, from this work perspective, have additional problems such as the biofouling onto filtration membranes, causing failures in the depuration performance. Among other reasons, biofouling is caused by *quorum sensing*-controlled biofilm formation on filtration membranes, so understanding keystone species in biofilm formation also represents an interesting topic for future research^36^. Thus, estimating the stability of the mentioned population would indicate a correct system function, so we propose monitoring this stability along time as a new control parameter on WWTP functioning. To quantify this stability, we calculated the difference between each sample and the centroid of their sampling year. Thus, we observed that in the warmer months populations tend to be more dissimilar, returning to baseline differences in the winter months. It is the effect we observed of the temperature on the population. Further analyses are needed to implement this monitoring measure, such as in the case of a problem detection in the plant checking how it would affect the dissimilarities of the populations. Monitoring with longer time series would help to verify the continued annual drift.

## Material and Methods

### Site description, samples collection and basic water characterization

Samples were collected over a period of two years from a full-scale WWTP with a membrane bioreactor system (MBR) located in Barcelona (Spain) and described in previous studies^37^. The 66 samples were taken monthly and consisted of collected mixed liquor from the three functional stages in which the bioreactor was divided (Supplementary Fig. S4). Physical-chemical parameters were measured in the influent (I.-) and the effluent (E.-) of the WWTP, according to standard methods: biochemical oxygen demand, BOD (UNE-EN-1899); chemical oxygen demand, COD (ISO-6060); ammonia, NH_4_-N (ISO-7150); total nitrogen, TN (ISO-11905); total phosphorous, TP (ISO-6878) and suspended solids, SS (UNE-EN-872). The removal rate (R.-) was calculated accordingly. Mixed liquor suspended solids (MLSS) were measured according to UNE-EN-872 and hydraulic retention time, HRT was also calculated. Detailed information concerning plant physical-chemical parameters is summarized in Supplementary Table S1.

### DNA extraction and 16S rRNA sequencing

Mixed liquor samples were analysed following a 16S metabarcoding strategy. Samples were stored at −80 °C until DNA extraction was performed using DNA Power Soil extraction kits. The V4 region of the 16S rRNA gene was amplified by PCR using the primers 515F (GTGYCAGCMGCCGCGGTAA) and 806R (GGACTACNVGGGTWTCTAAT). Libraries were prepared following the two-step PCR Illumina® protocol and these were subsequently sequenced on Illumina® MiSeq instrument (Illumina®, San Diego, CA, USA) using 2 × 300 paired-end reads^38,39^ and then it was analysed by amplifying and sequencing the 16S rRNA V4 gene using custom primers^40^.

### Sequence processing

DADA2 algorithm^41^ implemented in R pipeline^42^ was used to perform sequence analysis, such as denoise, filter, align pairs and filter out chimeras. This algorithm implements an error correction model that allows the differentiation of even a single nucleotide^21^, giving as a final output an amplicon sequence variant (ASV) table. A total of 3,454,577 good quality reads were obtained. The taxonomic assignment was performed using the naïve Bayesian classifier implemented in DADA2 using as reference database Silva (release 132), with a bootstrap cut-off of 80%^43^.

### Microbial diversity and statistical analysis

Microbial diversity and statistical analysis were performed using phyloseq, version 1.26.1^44^ and vegan, version 2.5.5^45^ R packages. Simpson index of diversity^46^ was calculated, per sample, as an estimation of community alpha diversity. Beta-diversity (differences between samples) was calculated using a Bray-Curtis dissimilarity matrix on Hellinger transformed data^47,48^ and permutational multivariate analysis of variance (PERMANOVA). Redundancy analysis (RDA) was conducted from the dissimilarity matrices to compress dimensionality into two dimensional plots, constraining physical-chemical information in the plot.

Predictive functional analysis based on 16S rRNA was performed using an adaptation of the Tax4Fun routine^49^ software package. To obtain the proportion of each community containing each specific function, we filtered a total of 57 KEGGs functional orthologues (Supplementary Table S2) within 5 pathways related to nitrogen and phosphorous metabolisms, biodegradation and quorum sensing^50–52^.

We also investigated the core microbial community, defining core community members as the ASVs occurring in the whole dataset, regardless of their abundance. The Bray-Curtis dissimilarity matrix of the core community table was computed and the distance of the beta diversity of the samples to their sampling year centroid was calculated using betadisper function (vegan package).

## Supplementary information

Supplementary information.

Supplementary information S2.

Supplementary information S3.

## Data Availability

Raw files are available in the National Center for Biotechnology (NCBI) repository under the project code PRJNA588045.
