# Supplementary material for: Tuning up microbiome analysis to monitor WWTPs’ biological reactors functioning
Source: Sci Rep. 2020 Mar 5;10:4079. doi: 10.1038/s41598-020-61092-1 (PMC7057949; doi:10.1038/s41598-020-61092-1)
Supplement: Supplementary file 1 — Supplementary information. [file 41598_2020_61092_MOESM1_ESM.docx]

**Tuning up microbiome analysis to monitor WWTPs' biological reactors functioning**

**Miguel de Celis^1^, Ignacio Belda^2^, Rüdiger Ortiz-Álvarez^3^, Lucía Arregui^1^, Domingo Marquina^1^, Susana Serrano^1^, Antonio Santos^1*^**

**
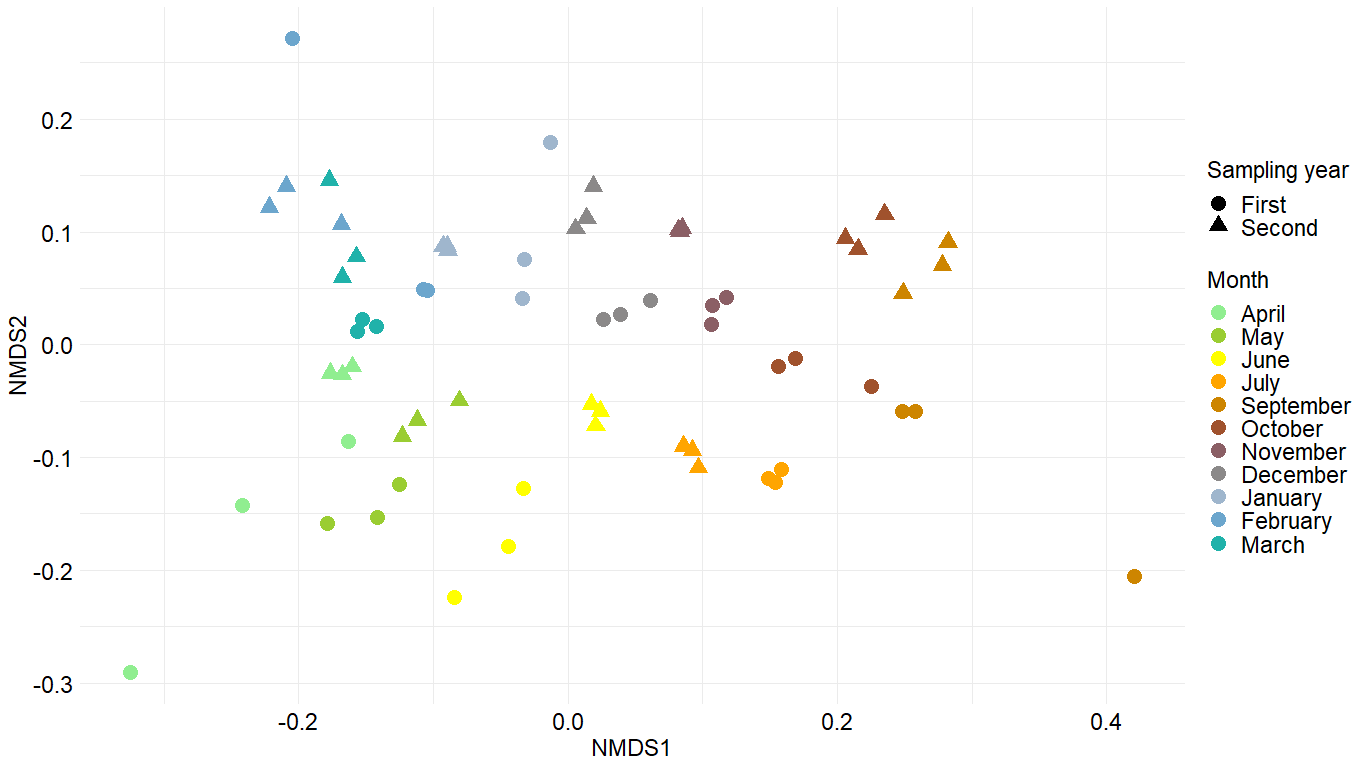
** **Supplementary Figure S1.** Ordination plot of bacterial community dispersion using non-metric multidimensional scale analysis (NMDS, stress = 0.151). The analysis was performed using the abundance table of ASV level and pairwise community distance was determined using Bray-Curtis. Dots and triangles correspond with each sample on the first or second sampling year, respectively. Colour indicates the corresponding month of the samples.

**
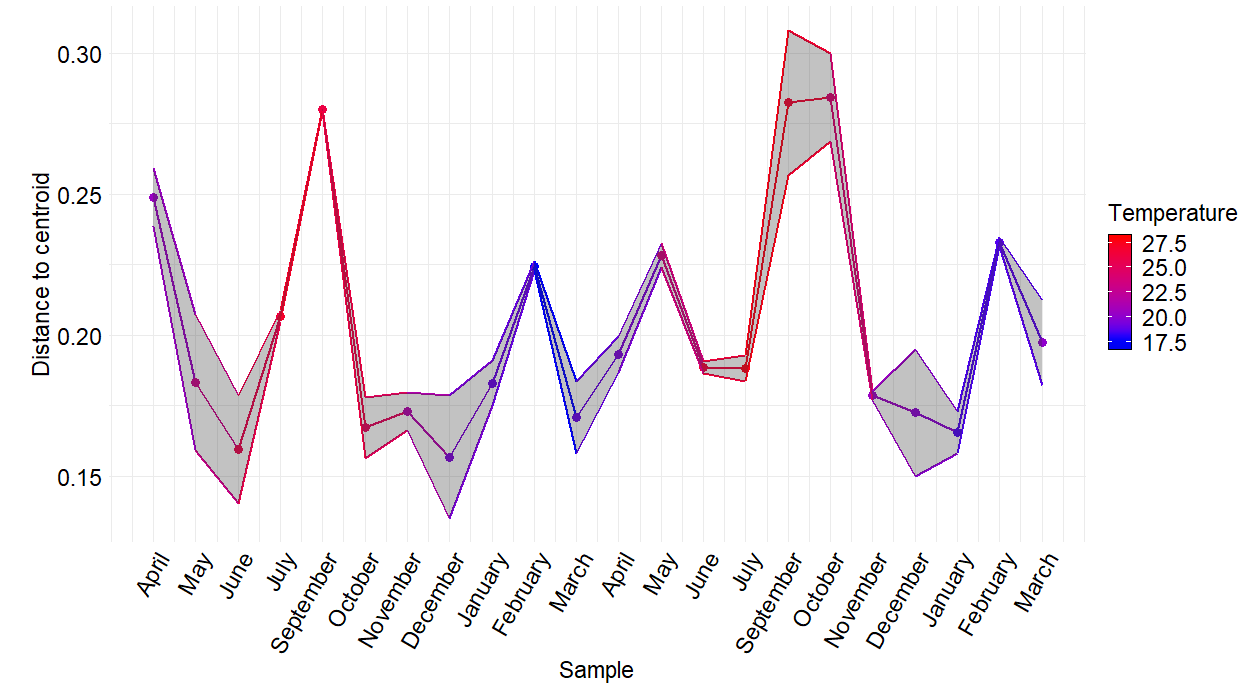
**

**Supplementary Figure S2.** Mean distance to centroid of bacterial population dissimilarities of samples taken on monthly. Each sampling year were considered independent, and both centroids were calculated. The colour gradient shows the average monthly temperature (ºC) on every sample.


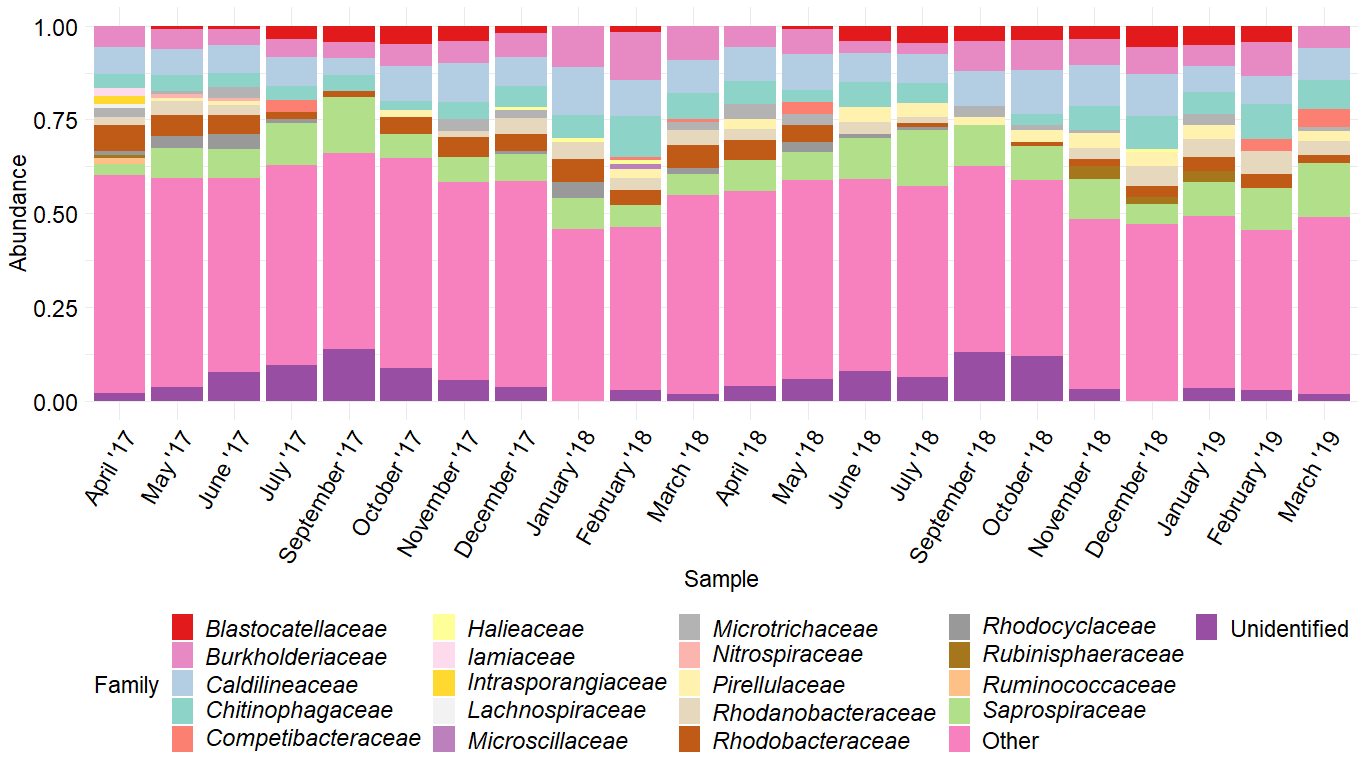


**Supplementary Figure S3.** Mean relative abundance of dominant bacterial family with a frequency higher than 2.5% on at least one sample. “Other”, includes phyla of frequency < 2.5%; and “unidentified”, taxonomically unassigned taxa. Sampling months are indicated, starting April 2017 and ending March 2019. There was no sampling in August.

***Influent***

***Effluent***

**Ax O-Ax Ax**

**MBR**

**Biological Reactor**

**Membranes**

**Supplementary Figure S4.** Flow chart of the studied MBR wastewater treatment plant. The three sampling points are the anoxic stage (Ax), the intermittent oxic-anoxic stage (O-Ax) and the anoxic stage (Ax). Physical-chemical parameters were analysed of influent and MBR effluent water.

**Supplementary Table S1.** Samples metadata. Detailed information concerning analyzed physical-chemical parameters and sampling information. Measurements units are included.

**Supplementary Table S2.** Functional predicted genes of interest. A total of 57 KEGGs within 5 pathways related to Nitrogen and Phosphorous metabolisms, biodegradation and quorum sensing.
